# Attention-Deficit/Hyperactivity Disorder And Inflammation: What Does Current Knowledge Tell Us? A Systematic Review

**DOI:** 10.3389/fpsyt.2017.00228

**Published:** 2017-11-09

**Authors:** Deepa Anand, Gabriela D. Colpo, Gregory Zeni, Cristian P. Zeni, Antonio L. Teixeira

**Affiliations:** ^1^Neuropsychiatry Program, Department of Psychiatry and Behavioral Sciences, McGovern Medical School, The University of Texas Health Science Center at Houston – UT Health, Houston, TX, United States; ^2^Federal University of Rio Grande do Sul, Porto Alegre, Brazil

**Keywords:** ADHD, attention-deficit, inflammation, inflammatory markers, cytokines

## Abstract

**Background:**

Attention-deficit/hyperactivity disorder (ADHD) is a complex condition that interferes with development and/or functioning. Our objective is to investigate the potential association between ADHD and inflammation.

**Methods:**

We conducted a systematic review of human studies measuring inflammatory markers in ADHD. The studies were identified by searching PUBMED, MEDLINE, EMBASE, PSYCHINFO, COCHRANE, and SCOPUS databases for peer-reviewed journals published until September 2016. We included cytokine gene expression and protein measured. Fourteen papers met the inclusion criteria.

**Results:**

Seven studies evaluated the association of cytokine gene polymorphisms in ADHD, and six studies measured cytokines levels in blood. One study analyzed the presence of cytokines in cerebrospinal fluid in patients with ADHD. Altogether, these studies indicate a possible role of inflammation in ADHD pathogenesis, despite the significant heterogeneity and contradictory results.

**Conclusion:**

Evidence points to the association of ADHD with inflammatory processes, but more studies are warranted.

## Introduction

Attention-deficit/hyperactivity disorder (ADHD) is a mental condition characterized by a persistent pattern of inattention and/or hyperactivity-impulsivity. ADHD symptoms must start before the age of 12 and be observed in more than one setting. ADHD can occur in 5% of children and 2.5% of adults, and is associated with significant impairment of social, academic, and occupational functioning across the lifespan (1).

Genetic and environmental factors have been postulated to be risk factors for ADHD (1–3). Strong genetic influence has been reported for ADHD, with 5–9 times increased risk in first-degree relatives of patients (4–6). Several authors have proposed association between ADHD and inflammatory mechanisms due to positive findings regarding inflammation-related genes (7–12). Cytokines have also been reported to play a pivotal role in tryptophan metabolism and dopaminergic pathways in the brain, which are also implicated in ADHD. Accordingly, it is conceivable that alterations in pro-inflammatory and anti-inflammatory cytokines may be influential in the pathogenesis of ADHD (10). Additionally, studies in rodents have shown that administration of cytokines like interleukin-1β (IL-1β), interleukin-2 (IL-2), and interleukin-6 (IL-6) can cause neurotransmission changes similar to those seen in ADHD such as increased norepinephrine and reduced dopamine levels (13, 14).

Among the risk factors for ADHD, preterm birth and perinatal infections are of significant relevance (15, 16). It is worth noticing that these conditions are associated with neuroinflammation marked by microglia activation. Microglia are the main resident immune cells of the brain. When activated, microglial cells release pro-inflammatory cytokines and other factors such as glutamate, contributing to neuroinflammation. Theoretically, a crosstalk between peripheral immune cells and microglia can potentiate inflammation both in the periphery and in the brain (17).

Although the association between ADHD and inflammation seems granted, the paucity of replication of genetic findings and longitudinal studies of inflammatory markers in ADHD prevents us to draw definite conclusions about the participation of inflammation in the pathogenesis of ADHD, or in the course and outcome (pathophysiology) of this condition. Due to the potential importance of the aforementioned association, we conducted a systematic review on the role of inflammation in ADHD. We will divide this review in genetic factors, and peripheral protein levels.

## Methods

A systematic review of the literature was performed according to the Preferred Reporting Items for Systematic Reviews and Meta-Analyses (PRISMA) statement. A literature search was conducted in PUBMED, MEDLINE, EMBASE, PSYCHINFO, COCHRANE, and SCOPUS databases using the keywords “ADHD OR attention deficit hyperactive disorder” AND cytokine OR interferon OR interleukin OR inflammation. The following limits were applied: published from 1946 to September 2016, humans, original articles, and English, Portuguese, and Spanish languages. Studies evaluating cytokine gene expression, protein production, or genetic polymorphisms were also included, provided they also met the previously mentioned inclusion criteria. Two independent reviewers conducted the search. The articles and their relevant reference citations were additionally reviewed.

## Results

**Figure 1:**
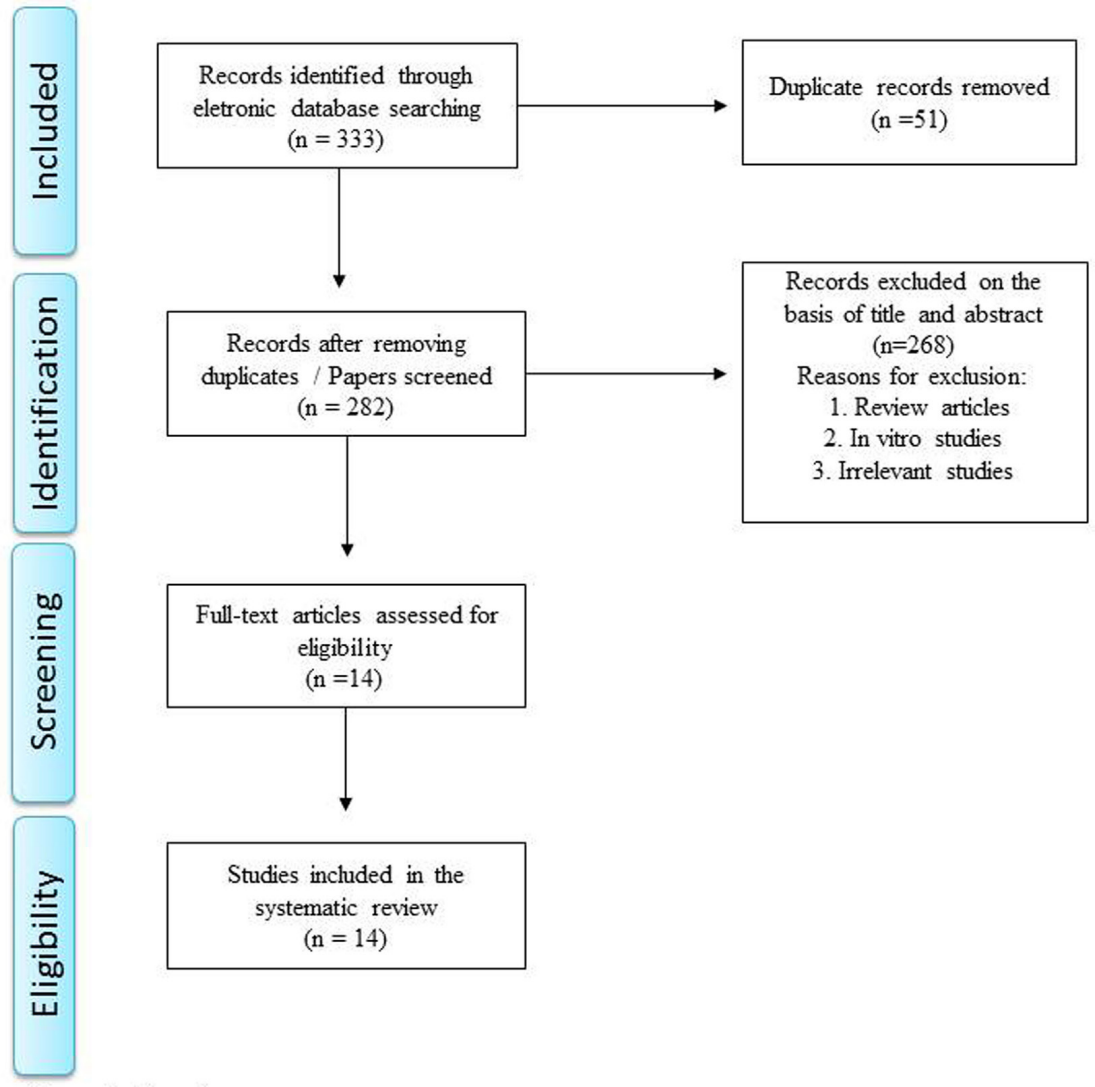
Flow diagram.

Literature search identified 76 articles in PubMed, 52 articles in Medline, 112 articles in EMBASE, 13 articles in PsycInfo, 80 articles in Scopus, and no articles in Cochrane. Manual review of all these references resulted in exclusion of those articles which were review articles, *in vitro* studies, irrelevant/unrelated studies, or duplicated studies. A total of 14 articles satisfied the inclusion and exclusion criteria and a summary of these studies is shown in Table 1. The specific number of included and excluded papers at each step is provided in a PRISMA flow chart (Figure 1).

**Table 1:** Studies measuring inflammation in ADHD.

Reference, country	Title	Samples	Assessments	Main conclusion
(10), Germany	Attention-deficit/hyperactivity disorder (ADHD) and glial integrity: S100B, cytokines and kynurenine metabolism—effects of medication	35 ADHD (24 treatment-naïve, 14 medicated)	IFN-γ, IL-1β, interleukin 2 (IL-2), IL-3 interleukin 6 (IL-6), IL-10, IL-13, IL-16, and TNF-α by ELISA	When compared to the control group, the interleukins IL-2, IL-6, IFN-γ, IL-10, IL-13, and IL-16 were higher in ADHD group and IL-1β lower in ADHD, although these were not statistically significant. The trend was reversed in ADHD medicated group
		21 controls		

(11), Germany	Attention-deficit/hyperactivity disorder (ADHD) and glial integrity: an exploration of associations of cytokines and kynurenine metabolites with symptoms and attention	35 ADHD (24 treatment-naïve, 14 medicated)	IL-1β, IL-2, IL-6, IL-10, IL-13, IL-16, IFN-γ, and TNF-α by ELISA	Statistically significant association between cytokines and ADHD symptoms. Increases in interleukins IL-16 and IL-13, were positively associated with hyperactivity and inattention, respectively; decrease in IL-2 was associated with opposition ratings in ADHD. In the CPT, IL-16 related to motor measures and errors of commission, while IL-13 was associated with errors of omission. Increased RT variability correlated with lower TNF-α, and higher IFN-γ levels
		21 controls		

(12), Germany	An exploration of the associations of pregnancy and perinatal features with cytokines and tryptophan/kynurenine metabolism in children with attention-deficit/hyperactivity disorder (ADHD)	35 ADHD (24 treatment-naïve, 14 medicated)	IL-1β, IL-2, IL-6, IL-10, IL-13, IL-16, IFN-γ, and TNF-α by ELISA	Increased IFN-γ was associated with lower birth weight and shorter pregnancy; increased IL-6 associated with paternal smoking; decreased TNF-α associated with obstetric problems
		21 controls		

(18), USA	Elevated blood levels of inflammation-related proteins are associated with an attention problem at age 24 months in extremely preterm infants	600 children born before 28 weeks gestation	25 inflammation-related proteins by multiplex detection system	Among children born extremely prematurely, recurrent or persistent elevations of IL-6, IL-8 and TNF-RI in blood during the first two postnatal weeks are associated with an attention problem at age 2 years

(19), Italy	Anti-Yo antibodies in children with ADHD: first results about serum cytokines	58 ADHD	IL-4, IL-6, IL-10, IL-17, TNFα, and IFNγ cytokine serum levels by ELISA	Higher levels of serum IL-6 and IL-10 detected in ADHD children than in controls
		36 controls		

(20), Iran	Effect of n-3 supplementation on hyperactivity, oxidative stress and inflammatory mediators in children with attention-deficit/hyperactivity disorder	103 ADHD children (6–12 years)	IL-6 levels by ELISA and C-reactive protein (CRP) by immune-turbidometrical	A significant reduction was observed in the serum levels of CRP and IL-6 after 8 weeks of supplementation therapy with n-3 fatty acids, which correlated with improvement in Conners’ Abbreviated Questionnaires scores

(21), USA	Cerebrospinal fluid cytokines in pediatric neuropsychiatric disease	42 ADHD	IL-2, IL-4, IL-5, IL-10, IFN-γ, TNF-α, TNF-β, TNF-β/LT by ELISA	90% of the ADHD children had detectable IL-2, 60% had detectable IFN-γ levels, 70% had detectable TNF-β levels, 62% had detectable IL-5, 7% had detectable IL-10
		22 SZ		
		24 OCD		

(22), USA	Angiogenic, neurotrophic, and inflammatory system SNPs moderate the association between birth weight and ADHD symptom severity	360 ADHD probands, 21 affected siblings, 17 unaffected siblings	A set of 164 SNPs from 31 candidate genes, representing five biological pathways	Reported that 2 SNPs in ciliary neurotrophic factor receptor (CNTFR) gene were associated with ADHD inattentive symptom severity. They also reported that SNPs in cytokine genes IL16 and S100B moderated the association between birth weight centile range and hyperactive-impulsive symptom severity

(8), USA	Genome-wide association scan of the time to onset of attention-deficit/hyperactivity disorder	958 ADHD proband–parent trios	Genotyping *via* SNP analysis	2 SNPs in IL-16 gene were associated with inattentive ADHD phenotype

(9), USA	Genome-wide association scan of quantitative traits for attention-deficit/hyperactivity disorder identifies novel associations and confirms candidate gene associations	958 ADHD proband–parent trios	Genotyping *via* SNP analysis	Nuclear factor Interleukin 3-regulated (NFIL-3) gene—C allele was associated with earlier onset ADHD

(23), Israel	Preferential transmission of interleukin-1 receptor antagonist alleles in attention-deficit/hyperactivity disorder	77 ADHD families	IL-1Ra gene variable number tandem repeat (VNTR) polymorphisms	IL-1Ra 4-repeat allele was associated with a significantly increased risk for ADHD, whereas the IL-1Ra 2-repeat allele was associated with a significantly decreased risk for ADHD
		86 ADHD probands and parents		

(7), Canada	Replication test for association of the IL-1 receptor antagonist gene, IL1RN, with attention-deficit/hyperactivity disorder	178 ADHD families	Genotyped by PCR using primers flanking the IL1RN intron 2 86-bp VNTR site	No evidence for association of IL-1Ra gene polymorphism with ADHD
		220 probands		

(24), Czech Republic	Clinical and molecular-genetic markers of ADHD in children	119 ADHD	Genotyped by PCR (DRD2, COMT, ACE, IL-6, CCR5, TNF-α, AGT, MAO-B, IL-2, BDNF, DRD4, DATI)	Reported statistically significant association of IL-6 and TNF-α gene polymorphism in ADHD
		153 controls		

(25), Spain	Association study of 10 genes encoding neurotrophic factors and their receptors in adult and child attention-deficit/hyperactivity disorder	546 ADHD	Polymorphisms for neurotrophins (NGF, BDNF, NTF3, and NTF4/5), a member of the cytokine family of NTFs (CNTF), and their receptors NTRK1, NTRK2, NTRK3, NGFR, and CNTFR	Study demonstrated association between cytokine family ciliary neurotrophic factor receptor (CNTF) and both adult and childhood ADHD
		546 controls		

### Cytokine Gene Polymorphisms in ADHD

ADHD is a highly heritable disorder and numerous molecular-genetic studies have been performed. The participation of genetic factors (heritability) in ADHD is high, around 70–78% (26). Our literature search found seven studies that evaluated the potential association of cytokine gene polymorphisms in ADHD.

Segman et al. evaluated the transmission of a variable number tandem repeat polymorphism at the interleukin-1 receptor antagonist (IL-1Ra) gene in 77 nuclear ADHD families (86 children with ADHD and their parents) (23). They reported increased transmission of the IL-1Ra 4-repeat allele (χ^2^ = 4.07, *P* = 0.04) and decreased transmission of the 2-repeat allele (χ^2^ = 4.59, *P* = 0.03) in children with ADHD. The IL-1Ra 4-repeat allele was associated with a significantly increased risk for ADHD (χ^2^ = 4.46, df 1, *P* = 0.035, RR = 1.292, 95% CI 1.01–1.66), whereas the IL-1Ra 2-repeat allele was associated with a significantly decreased risk for ADHD (χ^2^ = 4.65, df 1, *P* = 0.03, RR = 0.763, 95% CI 0.59–0.98). However, a larger study involving 178 ADHD families could not replicate this finding and did not show significant association between the IL-1Ra polymorphism with ADHD (7).

Another investigation addressed the association between ADHD and polymorphisms at IL-6 and Tumor Necrosis Factor alpha (TNF-α) genes in 119 children with ADHD and 153 healthy controls (24). A statistically significant difference in the allelic and genotypic frequency of the –174 polymorphism at the IL-6 gene was observed between the ADHD and control groups, with increased frequency of the IL-6 C allele in hyperkinetic children. In cognitive function tests, the 174 polymorphism (alleles A and G) at the IL-6 gene and the –308 polymorphism (alleles 1 and 2) at the TNF-α gene had statistically significant correlation with hand-eye test of the Neurobehavioral Evaluation System (NES2) that evaluates visual-motor coordination and accuracy sub-test of Shape Discrimination Test (TDT_P_) for assessment of attention, respectively (24). IL-6 AA homozygotes performed significantly better than individuals with IL-6 GG homozygotes in the hand-eye test of the NES2 battery. Similarly, individuals without any TNF-α gene allele 2 performed significantly better than individuals harboring allele 2 (homozygotes or heterozygotes) in the accuracy sub-test of TDT_P_ (24).

Lasky-Su et al. examined 958 parent-offspring trios (1,865 parents, 933 ADHD children, 87% males and 13% females; mean age of children with ADHD 10.88 ± 2.1 years) and found that two SNPs at the IL-16 gene were associated with inattentive ADHD subtype (*P*-value < 10^−5^) (9). Using the same study population, the authors conducted a genome-wide association study and found that the nuclear factor interleukin 3-regulated (NFIL-3) gene C allele was associated with earlier onset of ADHD (*P* = 0.002) (8).

Smith et al. evaluated the potential role of inflammatory system genes in moderating the association between birthweight percentile and ADHD symptom severity (22). This study involved a sample of 398 children (360 ADHD probands, 21 affected siblings, and 17 unaffected siblings; mean age 10.7 ± 3.02 years; 83% male and 17% females). Two SNPs at the Ciliary Neurotrophic Factor Receptor (CNTFR) gene were associated with the Conners’ Parent Rating Scale Inattentive score (CNTFR; *q* = 0.005 CNTFR; *q* = 0.021). They also reported that SNPs at the genes Interleukin-16 (IL-16) and S100B moderated the association between birthweight percentile range and hyperactive-impulsive symptom severity in children with ADHD (22).

Ribases et al. performed a population-based association study to evaluate the association between genes encoding neurotrophic factors and their receptors in adult and child attention-deficit/hyperactivity disorder (25). The sample included 546 patients with ADHD (216 adults and 330 children) and 546 gender-matched unrelated control subjects. One-hundred eighty-three single nucleotide polymorphisms covering 10 candidate genes were investigated. This study demonstrated association between the CNTFR gene and both adult (*P* = 0.0077, OD = 1.38) and child ADHD (*P* = 9.1e−04, OD = 1.40) (25).

### Cytokine Protein Levels and ADHD

Six studies reported measurements of peripheral cytokines in patients with ADHD (10–12, 18–20). Comparisons of cytokines serum levels between patients with ADHD and controls are shown in Table 2.

**Table 2:** Cytokines blood level comparison between ADHD patients and controls.

Cytokines	Results^a^	Reference
IL-1β	=	(10, 11)
IL-2	=	(10, 11)
	=	(19)
IL-4	=	(19)
IL-6	=	(10, 11)
	↑	(19)
IL-10	=	(10, 11)
	↑	(19)
IL-13	=^b^	(10, 11)
IL-16	=	(10, 11)
IL-17	=	(19)
TNF-α	=	(10, 11)
	=	(19)
IFN-γ	=^b^	(10, 11)
	=	(19)

*^a^Compared to controls*.

*^b^Post hoc analysis*.

In 2010, Oades et al. reported the serum measurements of IL-1β, IL-2, IL-6, TNF-α, interferon-gamma (IFN-γ), IL-10, IL-13, and IL-16 in 21 children with ADHD who were treatment-naïve (mean age: 8.9 ± 1.4 years) (10). These were compared to serum cytokine measurements in 21 controls (mean age 11 ± 1.5 years), 14 children who were receiving medication for ADHD (mean age: 12.6 ± 2.1 years), and 7 healthy siblings. No differences between the control group and the ADHD treatment-naïve group were found (10). The authors detected significant differences in the comparison of the children with ADHD who were receiving treatment, and the medication-naïve population. Lower levels of IFN-γ and IL-13 were seen in the ADHD medicated group (10). Furthermore, the ratio of TNF-α/IFN-γ was lower in the ADHD medication-naïve group when compared to the control group. However, the direction of the findings was reversed in the ADHD medicated group, i.e., the ratio of TNF-α/IFN-γ was increased when compared to the control group.

Using the same sample, Oades et al. further analyzed the correlation between cytokines and symptom ratings (inattention, hyperactivity, opposition, and anxiety) and continuous performance task measures (sustained attention, impulsivity, and variability). The authors reported statistically significant correlations between cytokines levels and ADHD symptoms (11). Increase in IL-13 was associated with increased inattention symptoms, high IL-16 was associated with increased hyperactive-impulsive symptoms and was positively associated with motor activity. Decrease in pro-inflammatory cytokine IL-2 was associated with more oppositional symptoms. Also, increased anti-inflammatory cytokine IL-16 and decreased in pro-inflammatory cytokines TNF-α and IL-6 were positively associated with commission errors. IL-13 and IL-16 were reported to have negative correlations with errors of omission in ADHD treatment-naïve and ADHD medicated group, respectively.

Oades et al. also conducted a very original study assessing the relationship between outcomes of inflammatory markers in children and adolescents with ADHD, and pregnancy/perinatal features (12). Increase in serum IFN-γ levels during childhood was associated with lower birth weight/shorter pregnancy. Increase in IL-16 levels was correlated with poorer infant health, and decrease in TNF-α levels was correlated with increased incidence of obstetric problems. Paternal smoking was associated with increased IL-6 levels in children with ADHD (12). Oades et al. also reported that maternal intake of supplements during pregnancy correlated with decrease in TNF-α and increase in IL-10 in children with ADHD (12).

In another study involving 103 children (age range 6–12 years) with ADHD, a significant reduction was observed in the levels of C-reactive protein and IL-6 after 8 weeks of supplementation therapy with omega-3 fatty acids. The decrease in cytokine levels was correlated with significant improvement in the Conners’ Abbreviated Questionnaires scores (20).

O’Shea et al. measured 25 inflammation-related serum proteins on postnatal days 1, 7, and 14 in 600 premature infants, born before 28 weeks of gestation (18). The investigators evaluated the potential association between inflammatory protein levels and behavioral problems, these latter assessed using parental responses to the Child Behavior Checklist for ages 1.5–5 at 2 years of age. Among the measured molecules, persistent or recurrent elevations of IL-6, TNF-RI, and IL-8 correlated with an increased risk of attention problems (18).

A more recent study provided further supports that the cytokine mediated inflammation is likely to be an important pathogenic factor in ADHD (19). Donfrancesco et al. compared 58 patients with ADHD (mean age: 9.5 ± 2.3 years), and 36 healthy controls (mean age: 9.6 ± 1.5 years). This study detected significantly higher levels of serum IL-6 and IL-10 levels in patients with ADHD (19). An intriguing finding in this study was that 45 of the ADHD children (77.6%) were positive to anti-Yo antibodies, autoantibodies that have activity against Purkinje cells of the cerebellum, whereas 34 of the control children were negative.

To best of our knowledge, there is only a single study which analyzed the presence of cytokines in cerebrospinal fluid (CSF) in patients with ADHD (*n* = 42) (21). It was reported that 90% of the children with ADHD had detectable IL-2 (mean: 1.65 ng/mL± 0.87), 60% had detectable IFN-γ levels (mean: 0.47 ng/mL ± 0.27), 70% had detectable TNF-β levels (mean: 2.45 ng/mL ± 0.60), 62% had detectable IL-5 (mean: 1.05 ng/mL ± 0.42), 7% had detectable IL-10 (mean: 0.29 ng/mL ± 0.09) and none had any detectable IL-4. No healthy controls were included in this study, making it difficult to draw inferences regarding the association with ADHD. However, the authors compared this finding to CSF cytokine profiles in obsessive-compulsive disorder and schizophrenia. They reported a relative increase in type 1 cytokines in CSF in obsessive-compulsive disorder compared to a relative preponderance of type 2 cytokines in schizophrenia, while children with ADHD had a CSF profile which was intermediate between these two (21). This study again lent credence to the possibility that immunological processes are likely involved in the pathogenesis of ADHD.

## Discussion

The current systematic review relied on data from 14 manuscripts and shows variable results regarding the association between inflammation and ADHD. Seven studies evaluated the possible association of cytokine gene polymorphisms in ADHD, six studies measured cytokines levels in blood and one study analyzed cytokines in CSF of patients with ADHD. Despite their significant heterogeneity, these studies indicate a possible role for inflammation in ADHD pathogenesis. It is worth mentioning that the heterogeneity of the molecules assessed in these different studies prevented us to perform meta-analyses.

Among the studies that evaluate immune-related genes, the results point to an association between increased risk for ADHD and enhanced/impaired inflammatory response. However, different genes were investigated, and the issue of results replication in psychiatric genetics was also noticed here. Segman et al. found that IL-1Ra 4-repeat allele was associated with a significantly increased risk for ADHD (23). This result was not replicated in a larger sample size (7). Ribases et al. performed a population-based study showing association between CNTFR and both adult and child ADHD (25). CNTF is a multifunctional neuropeptide that promotes survival and regulatory signals to neurons, astrocytes, and oligodendrocytes, and seems to be relevant in reducing damage during inflammatory responses. In addition, CNTF plays a key role during the development of the nervous system (27). As ADHD that can be conceptualized as neurodevelopmental disorder, it is possible a direct involvement of CTNF signaling pathways in the pathogenesis of ADHD. Besides this direct implication to ADHD, the polymorphisms of cytokine genes could be associated with ADHD risk factors (e.g., preterm birth and perinatal infection), leading to altered neuroinflammatory responses with subsequent impairment of the development of neural circuits.

The results on peripheral cytokines levels were also complex, but overall they suggest a low-grade inflammation in patients with ADHD. Interestingly, Oades et al. reported lower levels of IFN-γ and IL-13 in medicated patients with ADHD (10) when compared with medication-naïve patients, indicating the effect of treatment and/or clinical improvement on cytokine levels. As the levels of pro-inflammatory cytokines were also correlated with the severity of symptoms (11), one of the underlying mechanisms of this enhanced inflammation in ADHD could be the stress related-immune response. This hypothesis must be confirmed in longitudinal studies.

High levels of pro-inflammatory cytokines can influence synaptic plasticity and neurogenesis (28). Accordingly, cytokines can influence cognitive processes, including reaction time and working memory (29), that can be impaired in ADHD. Furthermore, the upregulation of pro-inflammatory cytokines such as IFN-γ and TNF-α modulates tryptophan metabolism. Studies have shown that tryptophan metabolites modulate several neurotransmitter systems, including dopaminergic transmission. Of note, lower levels of tryptophan and tryptophan metabolites are associated with the severity of ADHD symptoms (30). Pro-inflammatory cytokines can also activate microglia. When activated, microglia produce more pro-inflammatory cytokines that, in turn, promote activation of microglia, resulting in an inflammatory flow that further contributes to neuroinflammation and, theoretically, to the pathophysiology of ADHD (31). So far, no study has investigated microglial activation in the ADHD, an important aspect to confirm a direct pathogenic role for inflammation in ADHD.

There are several limitations in the literature addressing the role of inflammation in ADHD, including heterogeneous and small samples, lack of standardization of the biomarkers assessed, preventing comparisons among the studies. Even when the same molecule (IL-1Ra) was investigated by different studies, the results were not concordant. Accordingly, there is still much work to be done. Future studies must evaluate greater samples, including patients in different stages and across the spectrum of severity of the illness, and follow them prospectively with a representative panel of immune/inflammatory markers. This approach may determine a subgroup of patients in which inflammatory mechanisms play a meaningful pathophysiological role and, therefore, is more susceptible to immune-based strategies. In this context, an intriguing report needs to be replicated, i.e., high frequency of anti-Yo antibodies, an autoantibody known to impair cerebellar function (19). Regarding the frequent observation of altered balance and gait (signs of cerebellar dysfunction) in ADHD (32), it is tempting to hypothesize that the subgroup of patients with these motor signs would present higher levels of inflammatory markers than ADHD patients without motor signs.

In conclusion, over the past years, it has become recognized that inflammation may represent a common pathophysiological mechanism of major psychiatric disorders. Evidence also points to the association of ADHD with inflammatory processes. A better characterization of the involvement of inflammatory mechanisms in ADHD is warranted, and may reveal important aspects of the pathogenesis and psychopathology of ADHD, with the potential to effectively advance its treatment.

## Author Contributions

DA and GC contributed to perform the search, data collection, screening, and drafting the article. GZ helped writing the manuscript and CZ contributed writing and reviewing the manuscript. AT participated in the study design and the review of the manuscript. All authors have read and approved this final version.

## Conflict of Interest Statement

The authors declare that the research was conducted in the absence of any commercial or financial relationships that could be construed as a potential conflict of interest.

## References

[1] American Psychiatric Association. Diagnostic and Statistical Manual of Mental Disorders, F.E.A., VA, American Psychiatric Association. Washington: American Psychiatric Publishing (2013).

[2] Thapar A Cooper M Eyre O Langley K What have we learnt about the causes of ADHD? J Child Psychol Psychiatry (2013) 54(1):3–16. 10.1111/j.1469-7610.2012.02611.x 22963644 PMC3572580

[3] Thapar A Cooper M. Attention deficit hyperactivity disorder. Lancet (2016) 387(10024):1240–50. 10.1016/S0140-6736(15)00238-X 26386541

[4] Faraone SV Biederman J Monuteaux MC. Toward guidelines for pedigree selection in genetic studies of attention deficit hyperactivity disorder. Genet Epidemiol (2000) 18(1):1–16. 10.1002/(SICI)1098-2272(200001)18:1<1:AID-GEPI1>3.0.CO;2-X 10603455

[5] Elia J Devoto M. ADHD genetics: 2007 update. Curr Psychiatry Rep (2007) 9(5):434–9. 10.1007/s11920-007-0057-z 17915085

[6] Wallis D Russell HF Muenke M. Review: genetics of attention deficit/hyperactivity disorder. J Pediatr Psychol (2008) 33(10):1085–99. 10.1093/jpepsy/jsn049 18522996

[7] Misener VL Schachar R Ickowicz A Malone M Roberts W Tannock R Replication test for association of the IL-1 receptor antagonist gene, IL1RN, with attention-deficit/hyperactivity disorder. Neuropsychobiology (2004) 50(3):231–4. 10.1159/000079976 15365221

[8] Lasky-Su J Anney RJ Neale BM Franke B Zhou K Maller JB Genome-wide association scan of the time to onset of attention deficit hyperactivity disorder. Am J Med Genet B Neuropsychiatr Genet (2008) 147B(8):1355–8. 10.1002/ajmg.b.30869 18937294 PMC2605611

[9] Lasky-Su J Neale BM Franke B Anney RJ Zhou K Maller JB Genome-wide association scan of quantitative traits for attention deficit hyperactivity disorder identifies novel associations and confirms candidate gene associations. Am J Med Genet B Neuropsychiatr Genet (2008) 147B(8):1345–54. 10.1002/ajmg.b.30867 18821565

[10] Oades RD Dauvermann MR Schimmelmann BG Schwarz MJ Myint AM Attention-deficit hyperactivity disorder (ADHD) and glial integrity: S100B, cytokines and kynurenine metabolism – effects of medication. Behav Brain Funct (2010) 6:29 10.1186/1744-9081-6-29 20509936 PMC2889842

[11] Oades RD Myint AM Dauvermann MR Schimmelmann BG Schwarz MJ Attention-deficit hyperactivity disorder (ADHD) and glial integrity: an exploration of associations of cytokines and kynurenine metabolites with symptoms and attention. Behav Brain Funct (2010) 6:32 10.1186/1744-9081-6-32 20534153 PMC2900218

[12] Oades RD. An exploration of the associations of pregnancy and perinatal features with cytokines and tryptophan/kynurenine metabolism in children with attention-deficit hyperactivity disorder (ADHD). Atten Defic Hyperact Disord (2011) 3(4):301–18. 10.1007/s12402-011-0062-2 21785943

[13] Zalcman S Green-Johnson JM Murray L Nance DM Dyck D Anisman H Cytokine-specific central monoamine alterations induced by interleukin-1, -2 and -6. Brain Res (1994) 643(1–2):40–9. 10.1016/0006-8993(94)90006-X 7518332

[14] Anisman H Kokkinidis L Merali Z. Interleukin-2 decreases accumbal dopamine efflux and responding for rewarding lateral hypothalamic stimulation. Brain Res (1996) 731(1–2):1–11. 10.1016/0006-8993(96)00460-X 8883848

[15] Rand KM Austin NC Inder TE Bora S Woodward LJ Neonatal infection and later neurodevelopmental risk in the very preterm infant. J Pediatr (2016) 170:97–104. 10.1016/j.jpeds.2015.11.017 26707582

[16] Serati M Barkin JL Orsenigo G Altamura AC Buoli M. Research Review: The role of obstetric and neonatal complications in childhood attention deficit and hyperactivity disorder—a systematic review. J Child Psychol Psychiatry (2017). 10.1111/jcpp.12779 28714195

[17] Bhattacharya A Derecki NC Lovenberg TW Drevets WC. Role of neuro-immunological factors in the pathophysiology of mood disorders. Psychopharmacology (Berl) (2016) 233(9):1623–36. 10.1007/s00213-016-4214-0 26803500

[18] O’Shea TM Joseph RM Kuban KC Allred EN Ware J Coster T Elevated blood levels of inflammation-related proteins are associated with an attention problem at age 24 mo in extremely preterm infants. Pediatr Res (2014) 75(6):781–7. 10.1038/pr.2014.41 24614800 PMC4429865

[19] Donfrancesco R Nativio P Di Benedetto A Villa MP Andriola E Melegari MG Anti-Yo antibodies in children with ADHD: first results about serum cytokines. J Atten Disord (2016). 10.1177/1087054716643387 27095560

[20] Hariri M Djazayery A Djalali M Saedisomeolia A Rahimi A Abdolahian E. Effect of n-3 supplementation on hyperactivity, oxidative stress and inflammatory mediators in children with attention-deficit-hyperactivity disorder. Malays J Nutr (2012) 18(3):329–35. 24568073

[21] Mittleman BB Castellanos FX Jacobsen LK Rapoport JL Swedo SE Shearer GM. Cerebrospinal fluid cytokines in pediatric neuropsychiatric disease. J Immunol (1997) 159(6):2994–9. 9300724

[22] Smith TF Anastopoulos AD Garrett ME Arias-Vasquez A Franke B Oades RD Angiogenic, neurotrophic, and inflammatory system SNPs moderate the association between birth weight and ADHD symptom severity. Am J Med Genet B Neuropsychiatr Genet (2014) 165B(8):691–704. 10.1002/ajmg.b.32275 25346392

[23] Segman RH Meltzer A Gross-Tsur V Kosov A Frisch A Inbar E Preferential transmission of interleukin-1 receptor antagonist alleles in attention deficit hyperactivity disorder. Mol Psychiatry (2002) 7(1):72–4. 10.1038/sj/mp/4000919 11803448

[24] Drtilkova I Sery O Theiner P Uhrova A Zackova M Balastikova B Clinical and molecular-genetic markers of ADHD in children. Neuro Endocrinol Lett (2008) 29(3):320–7. 18580852

[25] Ribases M Hervas A Ramos-Quiroga JA Bosch R Bielsa A Gastaminza X Association study of 10 genes encoding neurotrophic factors and their receptors in adult and child attention-deficit/hyperactivity disorder. Biol Psychiatry (2008) 63(10):935–45. 10.1016/j.biopsych.2007.11.004 18179783

[26] Franke B Faraone SV Asherson P Buitelaar J Bau CH Ramos-Quiroga JA The genetics of attention deficit/hyperactivity disorder in adults, a review. Mol Psychiatry (2012) 17(10):960–87. 10.1038/mp.2011.138 22105624 PMC3449233

[27] Pasquin S Sharma M Gauchat JF. Ciliary neurotrophic factor (CNTF): new facets of an old molecule for treating neurodegenerative and metabolic syndrome pathologies. Cytokine Growth Factor Rev (2015) 26(5):507–15. 10.1016/j.cytogfr.2015.07.007 26187860

[28] McAfoose J Baune BT. Evidence for a cytokine model of cognitive function. Neurosci Biobehav Rev (2009) 33(3):355–66. 10.1016/j.neubiorev.2008.10.005 18996146

[29] Brydon L Harrison NA Walker C Steptoe A Critchley HD. Peripheral inflammation is associated with altered substantia nigra activity and psychomotor slowing in humans. Biol Psychiatry (2008) 63(11):1022–9. 10.1016/j.biopsych.2007.12.007 18242584 PMC2885493

[30] Aarsland TI Landaas ET Hegvik TA Ulvik A Halmoy A Ueland PM Serum concentrations of kynurenines in adult patients with attention-deficit hyperactivity disorder (ADHD): a case-control study. Behav Brain Funct (2015) 11(1):36. 10.1186/s12993-015-0080-x 26542774 PMC4636001

[31] Nakanishi H. Microglial functions and proteases. Mol Neurobiol (2003) 27(2):163–76. 10.1385/MN 12777686

[32] Buderath P Gartner K Frings M Christiansen H Schoch B Konczak J Postural and gait performance in children with attention deficit/hyperactivity disorder. Gait Posture (2009) 29(2):249–54. 10.1016/j.gaitpost.2008.08.016 18963991

